# Septicemic Outbreak in A Rainbow Trout Intensive Aquaculture System: Clinical Finds, Etiological Agents, and Predisposing Factors

**DOI:** 10.3390/life13102083

**Published:** 2023-10-19

**Authors:** Adrian Bălbărău, Larisa Maria Ivanescu, Gabriela Martinescu, Cristina Mihaela Rîmbu, Dumitru Acatrinei, Mircea Lazar, Iuliana Cocean, Silviu Gurlui, Alexandru Cocean, Liviu Miron

**Affiliations:** 1 Faculty of Veterinary Medicine, “Ion Ionescu de la Brad” Iași University of Life Sciences, Aleea Mihail Sadoveanu nr. 8, 700489 Iaşi, Romaniacrimbu@yahoo.com (C.M.R.); dacatrinei@yahoo.com (D.A.); lmiron@uaiasi.ro (L.M.); 2 Faculty of Physics, Atmosphere Optics, Spectroscopy and Laser Laboratory (LOASL), “Alexandru Ioan Cuza” University of Iasi, 11 Carol I Bld., 700506 Iasi, Romania; 3 Laboratory of Applied Meteorology and Climatology, A Building, Physics, Research Center with Integrated Techniques for Atmospheric Aerosol Investigation in Romania, RECENT AIR, “Alexandru Ioan Cuza” University of Iasi, 11 Carol I, 700506 Iasi, Romania

**Keywords:** *Oncorhynchus mykiss*, clinical examination, histopathological examination, bacteriological, water quality, real-time PCR

## Abstract

On the 23rd of September 2022, a small intensive aquaculture unit populated with rainbow trout (*Oncorhynchus mykiss*) reported increased mortality in adults and juvenile fish. The unit comprised 12 enclosed concrete basins with a capacity of ten cubic meters of water, populated with 150 kg of fish each. Fish were subjected to a clinical examination on the site, after which whole fish were harvested for a bacteriological and histopathological examination. Water quality parameters were examined using classic biochemical methods and Fourier Transform Infrared Spectroscopy in order to find out whether the environment in which the fish live is also a predisposing factor that could facilitate different pathogens and induce a state of disease in the fish. Real-time PCR was performed on strains of *Aeromonas* spp. sampled from the fish to accurately identify the pathogen species. The goal was to accurately identify the problems and predisposing factors that lead to disease outbreaks.

## 1. Introduction

Global aquaculture has reached record production in recent years, and fish farming will become increasingly important in the future as a means to provide food of animal origin. The total production of fisheries and the aquaculture sector, according to the Food and Agriculture Organization (FAO), a specialized agency of the United Nations, reached an all-time high in 2020, producing 214 million tons, of which 178 million comprised aquatic animals and the rest, 36 million tons, comprised algae. The amount destined for human consumption (excluding algae) in 2020 was 20.2 kg per capita, more than double the average of 9.9 kg per capita 50 years ago [1]. Also, in 1950, five years after the United Nations (U.N.) was founded, the world population was estimated to comprise around 2.6 billion people. Now, the total population of the earth is estimated at around 8 billion individuals, and the U.N. states that in the year 2100, there will be at least 11 billion people. That being said, any industry that can produce sustainable food has to grow and be more efficient than ever before if it is to be able to support the ever-increasing global need for food and nutrients. The total food production resulting from aquaculture is expected to grow by another 14% by 2030, and while this industry has the potential to offer food and nourishment to this planet’s increasing population, this growth must be sustainable [1]. It has also been stated in recent years that salmon and potentially other fish species require less feed in order to grow. It can be argued that salmon can convert 100 kg of dry feed into 65 kg of fillets compared to only 20 kg of poultry fillets or 12 kg of pork fillets [2]. If this proves to be the case, aquaculture might be the most sustainable way of producing food in the future.

In Romania, before 1989, trout aquaculture (both brown and rainbow trout) was the prerogative of the main managing authority of the forests (Romsilva Directorate) and of the national network of professional hunters and fishermen associations. Fish hatcheries belonged to the fishing associations managed by the two authorities and were subsidized by the state. After 1990, trout breeding developed intensively in freshwater aquaculture systems and was capitalized from the economic and touristic points of view in parallel with the species living freely in cold mountain waters. Year by year, the number of Romanian companies that had the objective of trout breeding increased, exceeding 360 in 2015. Romania is a net importer of fisheries and aquaculture products. In the past several years, the volumes have showed a slight upward trend, reaching a volume of 102,129 tons and €302 million in value by the end of 2019. There were 32 registered fish processing companies in 2019, which employed 1197 people (full-time equivalent), and the production amounted to 22,532 tons and was worth €87.5 million [3]. The total production in Romania of trout in 2019 was 2984.28 tons, of which 2690.07 tons was represented by rainbow trout [4].

In 1991, Fred P. Meyer claimed that diseases represent the largest cause of economic loss in aquaculture systems. In 1988 and 1989, farmers reported losses of millions of fish, attributed to different diseases [5]. Previous studies have shown that fish populations in Romania are affected by infectious diseases caused by bacteria belonging to the genera *Aeromonas*, *Vibrio*, and *Pseudomonas* [6].

Since then, this sector has grown, and it can be safely assumed that diseases are still a prevalent issue and the economic losses associated with them have also increased. Another problem is global warming, which is also affecting the aquaculture industry. Studies have shown an increase of about 0.6 °C up to the year 2000 since the pre-industrial age; in the future, the average global temperature is expected to rise by 2–6 °C by 2100. At first glance, these values may not seem large when compared with the changes normally occurring from day to day and throughout a year. The truth is that when considering the global average temperature, a change of 5–6 °C usually occurs between the middle of an ice age and the warm period between ice ages [7].

As ectotherms, fish are expected to be especially vulnerable to global warming. Chronic changes in temperature may represent a physiological load that can greatly reduce the ability of fish to cope with additional stress factors [8]. Adding to the problem, many diseases show increased virulence at higher temperatures as a result of reduced host resistance due to stress or a higher transmission rate [9]. For the welfare of farmed fish, the quality of water is also of extreme importance. Fish live in intimate contact with their environments through the significant surface area of their skin and gills, and it is well known that these animals are particularly vulnerable to inappropriate water quality [10].

Our goal is to identify problems that occur within this sector in Iași County and the Prut river basin, their cause, and predisposing factors. Using different methods of disease diagnostics and water quality parameter assessment, the goal is to correctly identify the cause of the disease outbreak and find out what pathogens pose a direct threat to the fish. Also, we are trying to find out whether the problems that occur in this particular unit are results of an improper facility design, an environmental problem, a specific pathogen, or a combination of the mentioned factors.

## 2. Material and Methods

**Sampling**

The fish sampled were raised in an intensive aquaculture unit that used an open (flow-through) water system and was composed of 12 concrete basins with a capacity of approximately ten cubic meters of water. There was only one type of species of fish grown in this particular unit, which was rainbow trout (*Oncorhynchus mykiss*).

**Fish facilities**

The fish were divided into basin by age and weight. The first basin contained the juveniles (up to 1 month of age) with an average weight of 10–15 g while other basins housed adults (8 to 9 months of age) which weigh between 400–600 g.

Fish were given approximately 10 g of feed per fish each day, which corresponded to 1 kg of feed for every 100 kg of fish.

The density in each basin was 15 kg of fish for each metric cube of water. The space was completely enclosed, with a constant indoor temperature of about 15 °C. 

**Figure 1:**
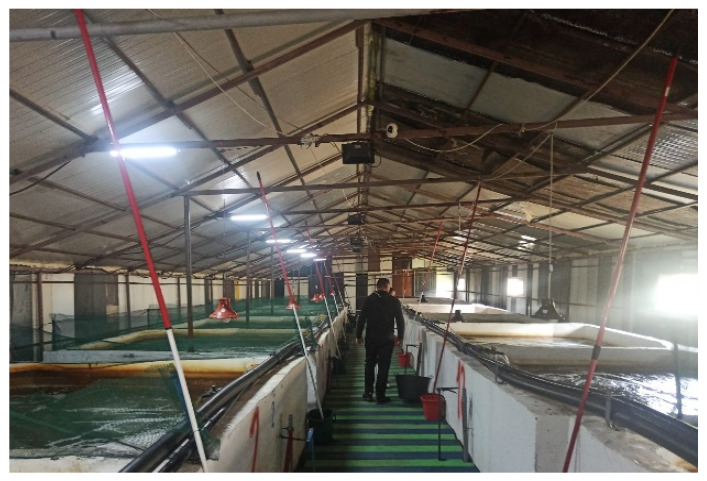
Intensive aquaculture rainbow trout farm.

The water came from an underground source with a depth of 120 m, but the point at which it was collected and then redirected towards the basins was located at a depth of 50 m (Figure 1).

Also, whole fish were sampled, preserved in ice, and used to extract not only blood from the hearts and livers but also samples from the surface of the skin to perform a bacteriological examination.

Water was also sampled from the source and the basins in clean glass containers and subjected to a physicochemical analysis.

**Methodology:**

The clinical examination followed Catherine A. Hadfield’s protocol [11] and started with an observation from a distance, and it was noticed that in all the basins, fish were active and alert and responded to tactile and visual stimuli. 

After this stage, the fish sampled underwent an external examination. The overall aspect of the skin and eyes was evaluated first, checking the coloration, fin abnormalities, or presence of external parasites. The clinical examination continued with the macroscopical evaluation of the gills, more specifically the color, integrity, and presence of potential parasites.

Fin and gill biopsies were harvested and subjected to a direct microscopical investigation in order to evaluate the presence/absence of parasites. The next step was an examination of the organs and internal cavities. The abdominal cavity was opened using a scalpel blade and the organs and intestinal mass were examined. In the case of the organs (spleen, liver, kidneys, heart), the color and consistency were examined while also looking for eventual abnormalities.

After this examination was concluded, liver samples were taken and subjected to a more detailed histopathological investigation. In order to perform the histopathological examination, the samples collected from the liver were fixed in formaldehyde 10% aqueous solution, embedded in paraffin, sectioned, and colored using the Masson trichrome method (H.E.A.). Masson’s Trichrome Stain Kit (G-Biosciences, St. Louis, MO, USA) was used as per the manufacturer’s protocol. Slides were placed in Bouin’s reagent at 60 °C for 1 h then cooled for 10 min. Equal parts of Weigert’s Reagent A and B were mixed and the slides were placed in the freshly prepared Weigert’s Hematoxylin reagent for 5 min. The slides were then rinsed with water, after which Briebrich Scarlet was applied for 15 min. Without rinsing, Phosphotungstic phosphomolybdic acid was added for 10–15 min. Aniline blue reagent was added for 5–10 min, after which Glacial acetic acid 1% was added for 3–5 min. The final steps involved dehydrating with ethanol, clearing, and placing a coverslip. The samples obtained were examined using a microscope, Leica DM 500 (Leica Microsystems Inc., Wetzlar, Germany).

The facility reported no problems for a period of 7 months, and after that, mortality rose, drastically reaching a recorded loss of 10 kg of fish each day. The juvenile fish presented higher mortality rates while the adults showed signs of inappetence or very low rates of feed consumption.

Water quality parameters measured on the spot using a Milwaukee MW605 MAX Waterproof Galvanic Dissolved Oxygen Meter (Milwaukee Instruments, Brookfield, WI, USA) were the dissolved oxygen and water temperature, after which dead or dying fish were sampled and subjected to a clinical examination.

The values of dissolved oxygen (DO) can vary, but the target is usually 6–15 mg/L, which corresponds to a 90% saturation for most species of fish [11]. It is also well known that food conversion efficiency is closely tied to the dissolved oxygen levels, and if the oxygen concentration is under 4–4.5 mg/L, this process slows down significantly. In general, at oxygen concentrations that are below 5 mg/L, growth and swimming performance may also be impaired [12].

The water turbidity was also high, based on the increased number of particles that obstructed the passage of light in the water. Water with low turbidity values is desirable for aquatic life; otherwise, fish gills might be affected [13], especially in the case of rainbow trout, which requires cold and crystal-clear water, high dissolved oxygen, moderate free carbon dioxide, proper water velocity, and balanced water discharge [14]. The excess unconsumed feed that was dissolved in the water seemed to form deposits on the surface of the gills that might hinder the fish’s capacity to use the dissolved oxygen. A high respiratory rate and an increased effort in breathing were observed in most cases and assessed based on opercular and gill slit movement and an increased gasping at the water surface. The temperature ranged between 11.6 to 11.9 °C and the oxygen values were between 8.3 and 8.5 mg/L.

The water parameters measured first were oxygen and temperature. After this determination, water samples were taken to the laboratory and other parameters were assessed using Boyd and Tucker’s protocol [15].

Quantofix kit (Macherey-Nagel, Düren, Germany) was used for semiquantitative determination of the NH_4_^−^, SO_3_^2−^, PO_4_^3−^, Fe^2+/3+^, and Cu^+/2+^ ions and the hardness. Spectroscopy (FT-IR) performed with Bomem MB154S spectrometer at an instrumental resolution of 4 cm^−1^ (Bomem, ABB group, Brampton, ON, Canada) provided information on the functional groups that have covalent bonds in organic and inorganic substances dissolved and/or dispersed in the basin and source water. The samples of basin water and water source were prepared and analyzed using FTIR spectroscopy following the procedures as described by Cocean et al. (2020) [16,17] and Garofalide et al. (2022) [18]. The pH was tested with the pH tester HI98103 from Hanna Instruments Inc., Woonsocket, Rhode Island, NW, USA.

**Microbiological testing**

The microbiological examination was carried out on the trout that showed lesions. Two specimens were taken from the pools where the mortality had occurred. The trout were necropsied and biological samples were isolated from the organs with lesions (liver) and heart. The diagnostic steps consisted of taking the biological material under aseptic conditions and seeding on the usual culture media: Mueller Hinton Nutrient Broth (Oxoid) and Mueller Hinton with horse blood Agar (Oxoid). The samples were incubated at 35 °C for 24–48 h. The strains isolated in pure culture were examined microscopically to determine cell morphology using Gram stain. To determine the metabolic properties, all bacterial strains were seeded on special culture media: Eosin Methylene Blue Agar Medium (Levin Agar, Oxoid), Thiosulfate– Citrate Bilsaltsucrose Agar (TCBS Agar, Oxoid), and Simmons Citrate Agar (Merck, Merck KgaD, Darmstadt, Germany).

Also, the oxidase assay (detection of the enzyme cytochrome oxidase) was performed, which is indicative of the classification of Gram-negative bacteria. The isolated strains were tested using the API20E and API 20NE biochemical test systems (BioMerieux, Craponne, France). Based on the similarity of the metabolic profile, the isolated germs were identified.

After that, species confirmation was performed using matrix-assisted laser desorption/ionization time-of-flight mass spectrometry (MALDI-TOF, Bruker MALDI Biotyper^®^ Sirius, Billerica, MA, USA). The working technique consisted of using a 96-well Maldi-Bruker metal plate, on the surface of which the same type of colony was distributed in three different wells, over which 1 µL of 70% formic acid and, after drying, 1 µL of HCCA (α-cyano-4-hydroxycinnamic acid) matrix solution were added. After drying, the plate was placed in the MALDI-TOFF analyzer to perform ionization mass desorption and time-of-flight detection of ions from the support matrix. Depending on the ratio of specific mass to ionic charge, the specific protein profile of each analyzed microorganism was determined.

**Molecular testing:**

DNA extraction was performed from four bacterial cultures using the PureLink Genomic DNA Mini Kit (Thermo Fisher Scientific, Waltham, MA, USA) according to the manufacturer’s protocol. qRT-PCR was performed using NZYTech Real-time PCR Kit for detecting *Aeromonas salmonicida* genomes. PCR amplification was performed in a C1000™ Thermal Cycler (Bio-Rad, Hercules, CA, USA) with CFX96™ Real-Time Detection System. The primer and probe mix provided exploits the TaqMan principle using the aopP gene located on A. salmonicida plasmid pAsal1 [19]. The reactions were performed using the standard protocol for qRT-PCR according to the manufacturer’s protocol. Reactions’ final volume was 25 µL (5 µL of extracted DNA was added to a 20 µL reaction mixture). In addition, endogenous control reaction was performed to provide information regarding the quality of the biological sample. Fluorogenic data were collected through FAM and VIC channels.

## 3. Results and Discussion

**Figure 2:**
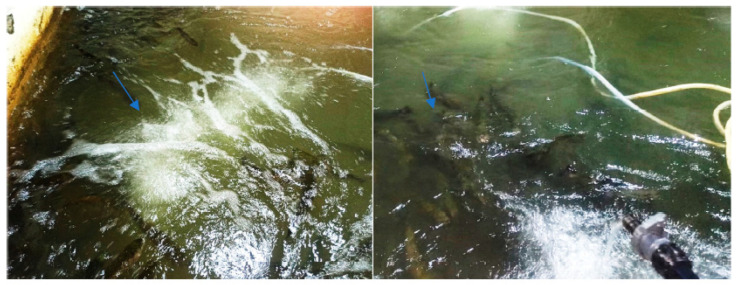
Fish gathered in the vicinity of the air pump and near areas of turbulence.

At first, when feed was administered, few fish changed their behavior, and in all basins, they gathered around the air pumps or close to other areas of turbulence, indicating a possible lack of oxygen or the inability to use the oxygen in the water (Figure 2).

In the case of the clinical examination, the skin, eyes, and fins appeared normal without any light or dark spots, hyperemia, ulcers, erosions, or visible parasites. The gill examinations showed areas of necrosis and gill hemorrhage, no visible parasites, and deposits of reddish small particles on the gill surfaces. These deposits were coming from unconsumed feed and were reducing gill functionality, which could explain the fact that even if, for this species, the values of the temperature and oxygen detected in the water appeared to be the desirable ones, they still had an apparent difficulty when breathing.

**Figure 3:**
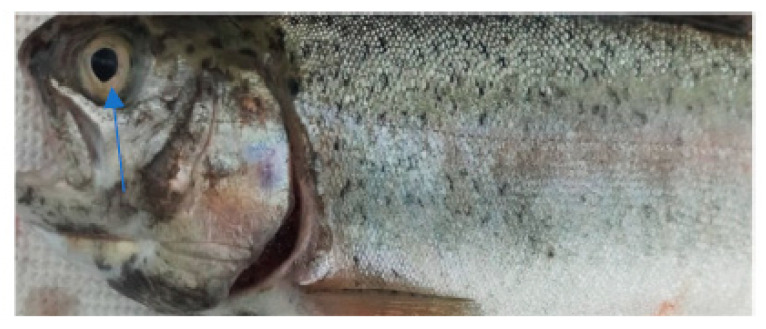
Clinical examination of the eyes.

**Figure 4:**
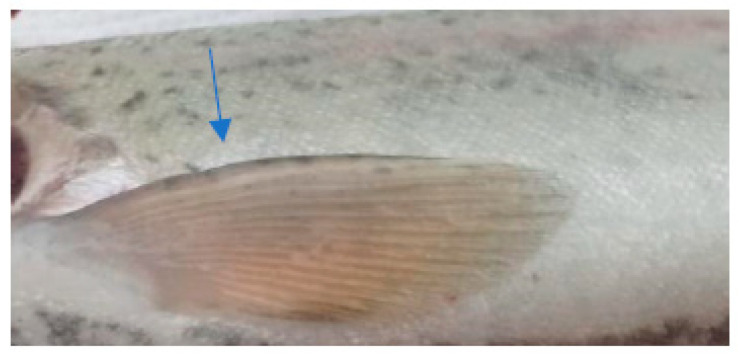
Clinical examination of the pectoral fins.

**Figure 5:**
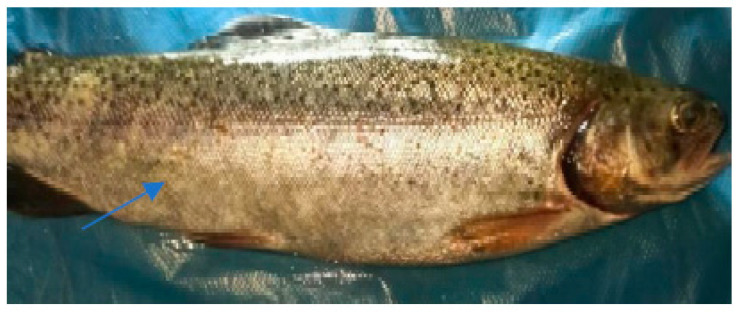
Overall assessment of the body.

**Figure 6:**
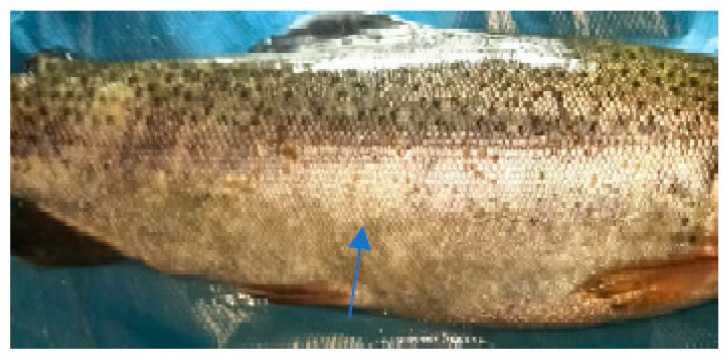
Clinical examination of the skin.

**Figure 7:**
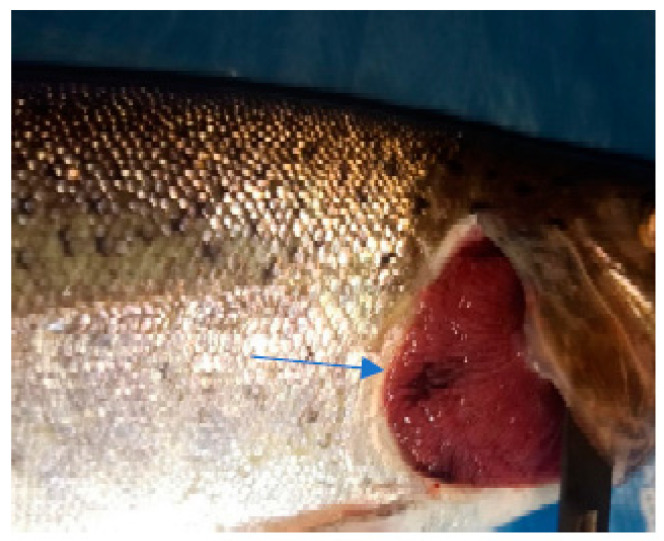
Gill hemorrhage and small areas of necrosis (blood and necrotic tissue on the gill surface).

**Figure 8:**
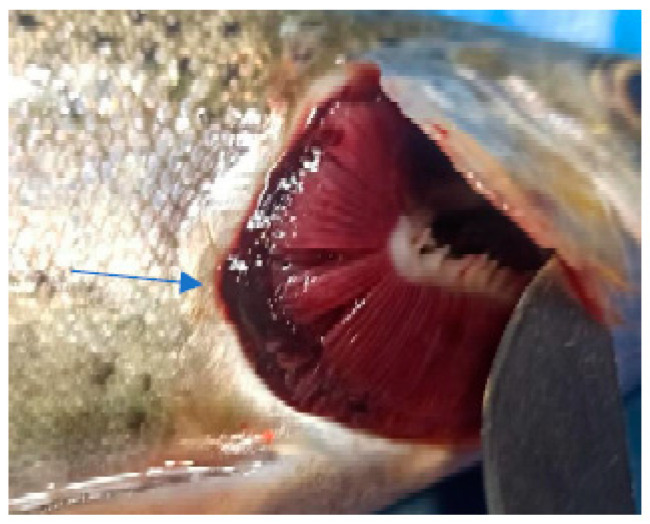
Gill hemorrhage (blood present between the gill arches).

The clinical examination of the eyes showed no modifications, which meant that the fish had died recently, and no post mortem modification could have altered the further examination. The overall assessment of the body, skin, and fins showed no visible lesions, which indicates that the mortalities were the result of a disease with a superacute evolution (Figure 3, Figure 4, Figure 5 and Figure 6). The only external lesions seen at the clinical examination were gill hemorrhages and small areas of necrosis on the gill tissues, which could have been self-induced or a consequence of predation (Figure 7 and Figure 8).

**Figure 9:**
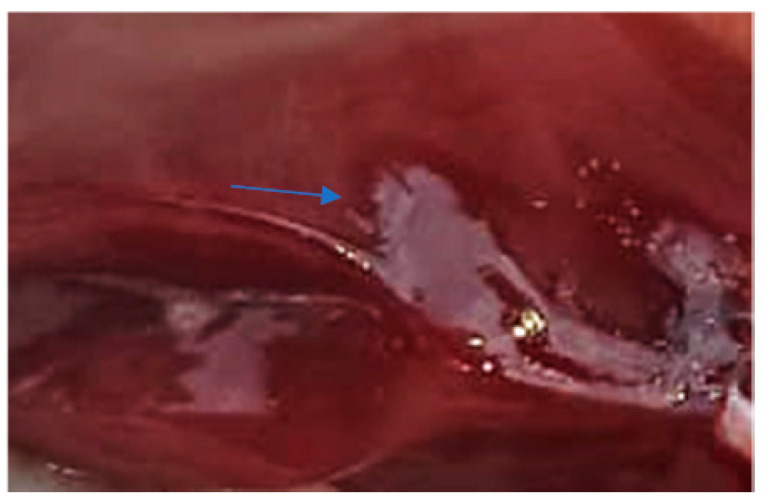
Red areas of congestion on the liver surface (dark-red spots on the organ’s surface).

**Figure 10:**
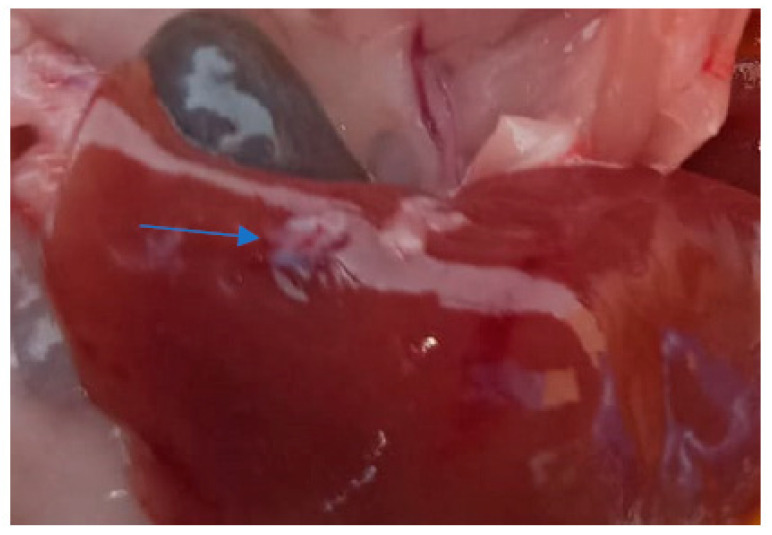
Necrosis area (scar tissue).

The heart, spleen, and kidney appeared normal macroscopically with no color modifications and normal consistency. The liver in all the examined subjects had signs of congestion with dark-red spots on the surface (Figure 9) and what appeared to be a very brittle consistency. Also, in the case of one adult, an area of scar tissue was observed, most likely induced by a necrotic lesion (Figure 10). In one juvenile, the liver had a yellowish color that indicated possible steatosis [20].

**Figure 11:**
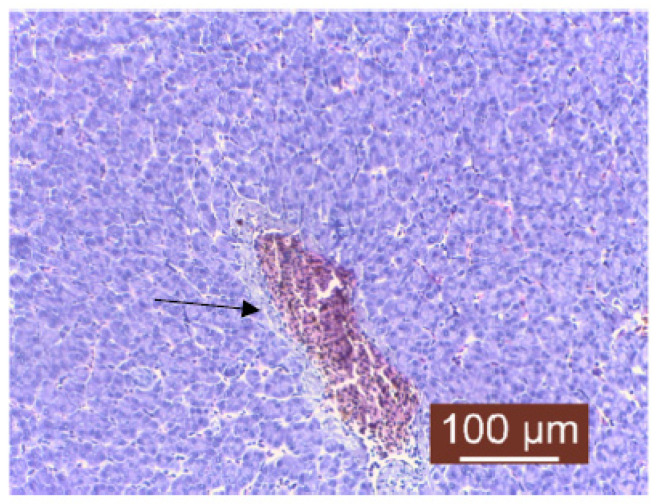
Liver: moderate interstitial congestion (Masson’s trichrome stain).

**Figure 12:**
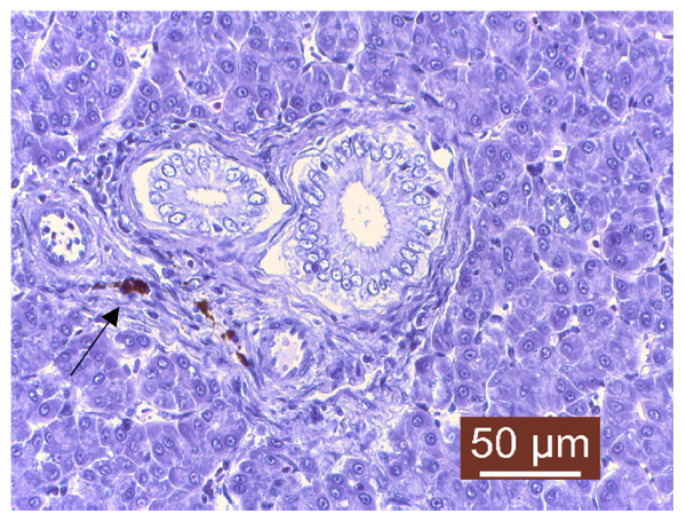
Liver: hemosiderosis near bile ducts (Masson’s trichrome stain).

**Figure 13:**
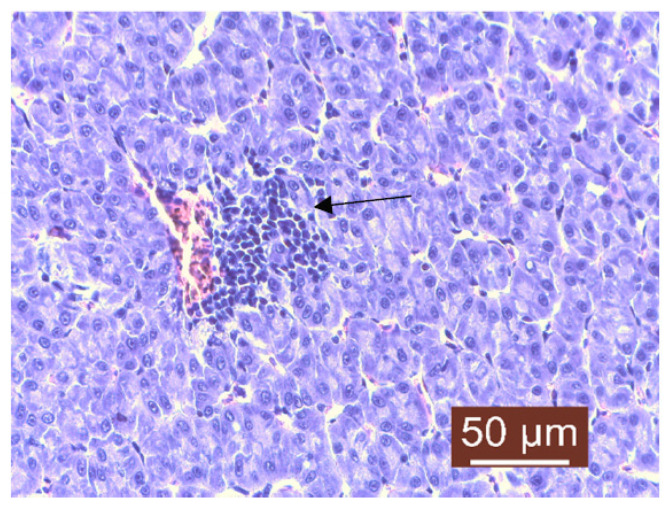
Liver: lymphocyte infiltrate near blood vessel (Masson’s trichrome stain).

**Figure 14:**
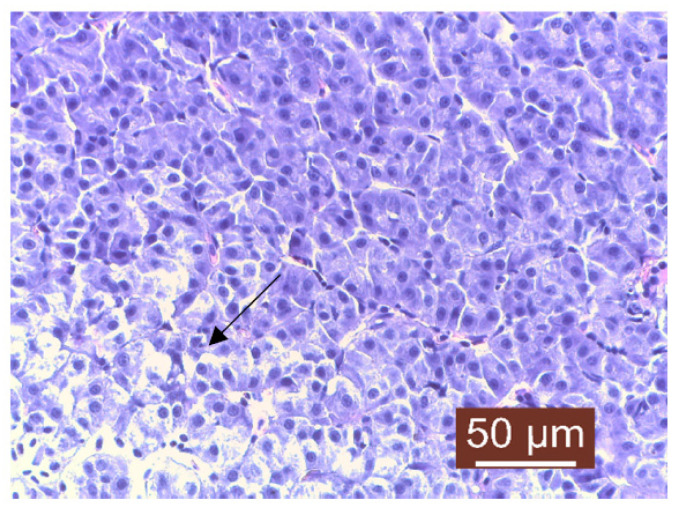
Liver: necrosis of hepatocytes (Masson’s trichrome stain).

**Figure 15:**
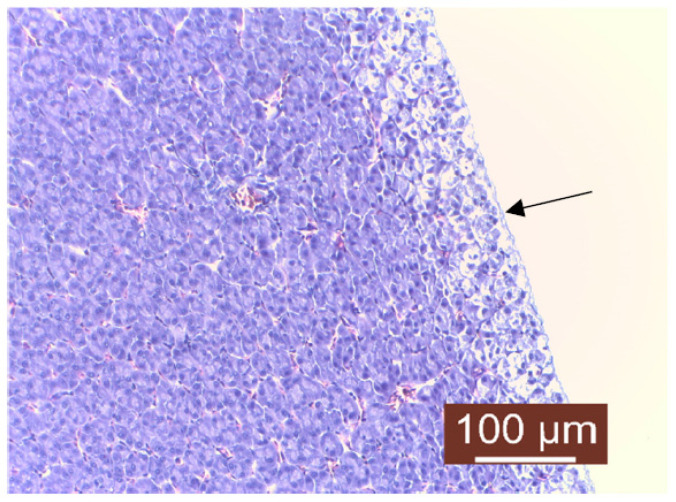
Liver: subcapsular necrosis (Masson’s tricchrome stain).

**Figure 16:**
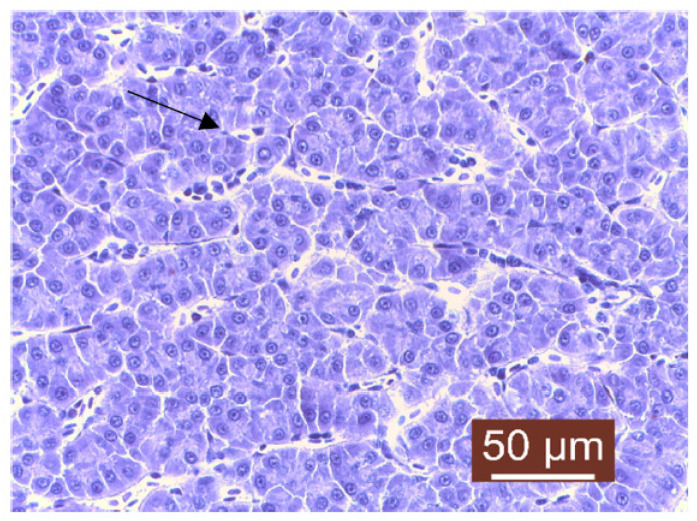
Apparently normal area of the liver with a slight increase in triglyceride content (Masson’s trichrome stain).

The histopathological examination of liver samples showed congestion (Figure 11), hemosiderosis (Figure 12) an increased number of lymphocytes in the liver (Figure 13), and necrosis in the liver mass including the subcapsular area (Figure 14 and Figure 15). Fish hepatocytes are normally more vacuolated than those of mammals, containing a relatively higher lipid and/or glycogen concentration [21]—a fact that may explain the slightly higher content of triglycerides (Figure 16).

The coloration, texture, and quantity of hepatocyte cytoplasm in each fish may also vary, and can be influenced by factors such as species, age, gender, nutritional status, or the effects of different toxic or inflammatory diseases [22]. The liver congestion observed was examined while also taking into account the degree to which each fish was exsanguinated at sacrifice and the amount of care taken to not manually squeeze the liver sample during necropsy. Congestion of the hepatic tissue may have been a result of sampling since the organ was already very fragile; a partially autolyzed liver or a focally traumatized one during sampling may result in the misdiagnoses of hepatocellular necrosis [23], but since lesions of necrosis were slightly visible with the naked eye on the surface of the liver, there was a suspicion of hepatocellular necrosis associated with possible inflammation and a dietary imbalance in vitamin E or selenium [24], or it could have been the result of a toxic disease, the latter possibly being induced by the level of chemical pollutants detected during quality measurements of the water. Hemosiderosis is characterized by an excessive deposit of hemosiderin (yellow–brown pigment) in the tissues of vertebrates (Figure 12). This process usually follows hemorrhage from trauma, chronic congestion, hemolytic disorders, parasitic infestation, or exposure to toxic chemicals [25]. Some studies have shown a higher occurrence of hepatocellular hemosiderosis in several species of fish, which was later linked to chemical pollution [18,26]. Lymphocyte infiltration is the first line of defense that responds to cell death and may indicate exposure to potential pollutants. Lymphocyte infiltration can also be linked to other changes, but, in general, the main objective is to neutralize and destroy the etiological agent, clean the tissue, and remove any dead cells to allow the proper recovery of the damaged tissue [27]. Hepatic lipidosis or steatosis is a condition characterized by excessive fat accumulation within the hepatocytes. It was observed only macroscopically in the case of one juvenile fish, and for this diagnostic to have a meaning linked to the fish’s health status, steatosis should be treated as a degenerative condition that is detrimental to the host. Even if studies in fish on this subject are somewhat limited, like in the case of mammals, the excessive storage of lipids within the liver may cause oxidative stress that can damage the cellular structures [28]. It is known that steatosis is induced by environmental contaminants such as organochlorine insecticides [29].

Further investigations are required to reach a solid conclusion regarding hepatic steatosis in this case, but factors such as nutrition and water toxicity may play major roles.

The inhomogeneous pH of the water from the source (spring) indicates the presence of compounds that were slightly miscible with water and that were both basic and acidic in nature (Table 1). They may even have been amphoteric compounds and even amino acids. Compounds from the water source were detectable in the pool water, with an increase in the concentration of iron ions. There was also a precipitation (coagulation) of red particles specific to iron oxide. Although SO_4_^2−^ ions were not analyzed due to the lack of a reagent, the presence of SO_3_^2−^ ions in an unusual concentration in general in water (5 mg/L) either indicates contamination with water cleaners (detergents) or is due to other causes, but contamination at the water source is also possible. Ammonia is a by-product of protein metabolism and is excreted by fish or results after the bacterial decomposition of organic matter such as wasted feed or feces. In the case of “total ammonia”, the desirable values seemed to be below 1 mg/L [30]. The phosphate level desirable for fish culture is 0.06 mg/L [31], but other sources indicate an accepted level of up to 1 mg/L. The toxicity of sulfite ion (SO_3_^2−^) and its lethal potential were studied in the past and were estimated to occur at concentrations of 180–320 mg/l, 100–3200 mg/L, and 180–320 mg/L. These values are quite different from each other, possibly because the pH value of the solution and the concentration of sulfite ions are difficult to keep constant during testing. Later, it was proven that the toxic potential of the sulfite ion for fish is strongly influenced by pH, and a value of 64 mg/L was established to be detrimental to the fish at a pH value of 6 [32]. That being said, it can be assumed that a value lower than 64 mg/L is desirable. Phosphate (PO_4_^3−^) and ammonium (NH_4_^−^) ions also exceeded the permitted limits by up to 10 times, and the contamination seemed to be also at the water source. The cause could have been fertilizers, household water, or other infiltrations. The Phosphate (PO_4_^3−^) and ammonium (NH_4_^−^) levels in the basins might have been indicators of a high concentration of organic matter (wasted feed or feces). The copper (Cu^+/2+^) values were below 0.1 mg/L and did not pose a problem. That being said, studies have shown that exposure to low (0.18 mg/L), medium (0.56–1.0 mg/L), or high levels (3.2 mg/L) of copper ions (Cu^+/2+^) can induce morphological changes in some fish [33].

The total hardness is very high, and even if the value is above the desirable range of 50–150 mg/L, as CaCO_3_ [31], it usually does not pose a problem, but may indicate the presence of insoluble substances that can be dangerous, especially in the quantities indicated by concentrations above 4.5 mol/m^3^. Also, water with a total hardness that is above 200 mg/L may be unresponsive to fertilization, and a high pH associated with an increased calcium concentration may lead to the rapid precipitation of calcium phosphate [34].

It is recommended to repeat the analyses periodically (at intervals of 3–6 months). In this regard, analyses of the samples of water collected from the same locations (basin and source) were repeated on the 28th of March 2023, and they showed values comparable to those from the 23rd of September 2022, with a slight mitigation of ammonium ions contained in the water source, as stated in Table 2.

**Figure 17:**
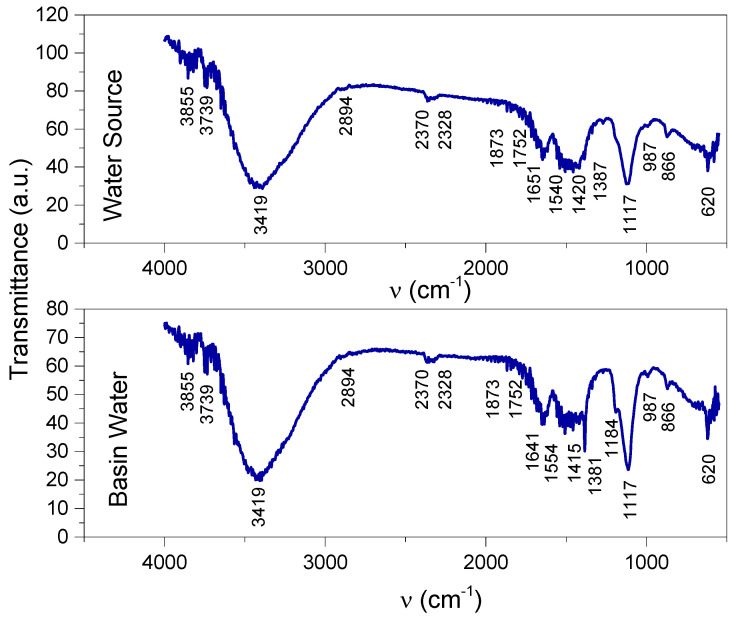
FTIR spectra of samples “Basin Water” and “Water Source”.

The FTIR spectra of the two samples of water from the basin and source (Figure 17) provided information regarding functional groups of organic and inorganic origin, as stated in Table 3.

The ions detected using Quantofix kit tests were confirmed by the FTIR spectra. Amino salts are evidenced by the bands at 3419 cm^−1^ and 1554/1540 cm^−1^ in the spectra of the water samples collected from the basin and source, these bands being also assigned to amines and amides [35,36,37,38].

The 1420 cm^−1^ and 1182/1117 cm^−1^ vibrations, assigned to SO_2_ stretching asymmetrically and symmetrically, respectively, confirm the SO_3_^2−^ groups detected using the Quantofix kit, denoting also sulfones and sulfoxides [17,35,36]. Phosphates indicated by the Quantofix tests were also evidenced by the FTIR spectra, with vibrations at 1182 cm^−1^ in the basin sample and 987 cm^−1^ in both basin and source samples [36]. The band at 1182 cm^−1^ is also assigned to organic compounds with phosphorus [36].

Hydroxyl groups, free and bonded, assigned to the stretching vibrations at 3855 cm^−1^, 3739 cm^−1^, and 3419 cm^−1^, denote alcohols, carboxylic acids (together with the vibrations in the ranges of 1554–1415/1540–1420 cm^−1^), and inorganic compounds such as Mg(OH)_2_, Ca(OH)_2_, and Fe_2_(OH)_3_/Fe(OH)_2_ or silanol groups in organic compounds (together with the bands at 1117 cm^−1^) [36,39,40,41,42].

Nitrites, nitrates, and nitro organic compounds were evidenced in the FTIR spectra by the vibration bands at 2328 cm^−1^ and 1641/1651 cm^−1^ and also in the ranges of 1554–1415/1540–1420 cm^−1^ and at 1381/1387 cm^−1^ and 866 cm^−1^. Diazo compounds (2328 cm^−1^ [16,36]) and aldehydes (1651 cm^−1^ and 1381/1371 cm^−1^ [36], in the same range as nitro compounds and nitrates) were also evidenced in the two spectra.

The IR vibrations indicated both aliphatic and aromatic compounds (2894 cm^−1^ for aliphatic and 1554–1415/1540–1420 cm^−1^ and 866 cm^−1^ for aromatic [36]; CH aromatic compounds were expected to be in the range of 3080–3030 cm^−1^ [36], overlapping with the wide band with the peak at 3419 cm^−1^).

The similarity between the two spectra shows that the chemical compounds originated in the source of water. It is worth noting that the decomposition of the nitro organic compounds with large molecules into small molecules and nitrates is evidenced by the sharp peak at 1381 cm^−1^ in the basin spectrum while its corresponding peak in the source spectrum (at 1387 cm^−1^) was of lower intensity and part of the wider band in the range of the 1540–1420–1387 cm^−1^ wavenumbers.

The chemical species evidenced using Quantofix kit tests and with FTIR spectroscopy showed a high level of pollution that had been generated right from the water source. In turn, the water source was under the influence of pollution factors, both in the atmosphere (through rainwater) as well as on the ground (fertilizers and other substances used in agricultural treatments administered excessively), in addition to those resulting from the burning of vegetable waste, household waste, and the discharge of wastewater [16,17]. Chemical contamination represents a major risk for the life of the fish in the pool through toxicity that can directly cause illness and even the death of the fish (directly and/or indirectly) through a decrease in immunity and exposure to various pathogens, as was true in the case studied by the present work. The fact that the pollution of the pool with chemical compounds had been induced by the water source itself indicates the need for measures to eliminate or reduce these pollutants to a minimum level such as changing the water source if possible or using methods of filtering/treating the water at the entrance in the basin.

**Results of the microbiological examination**:

For the bacteriological examination, fish that showed the most pronounced clinical signs were sampled from three different basins. From a series of samples, the bacteriological exam was performed using samples from the liver and resulted in a mixed culture of *Enterobacter (Cronobacter) cloaceae* and *Pseudomonas* sp. *Enterobacter cloaceae* belongs to the *Enterobacteriaceae* family and can be noticed in a multitude of environments such as human or animal feces, water, soil, plants or plant materials, insects, and dairy products [43,44]. Species of the Enterobacter cloacae complex are widely encountered in nature, but they can act as pathogens [45]. Resistance to broad-spectrum antibiotics and occasionally to last-resort carbapenem has led to an increased interest regarding this particular group of organisms [46]. The excessive use of antibiotics in fish farming has also played a role in the appearance of resistant strains that can lead to several health problems [47]. In the case of *Pseudomonas* sp., further investigations were preformed to accurately determine the species; the results of studies show that in the case of salmonids, species of *Pseudomonas* can induce clinical signs such as petechial hemorrhages, both in the skin as well as in the peritoneum and liver [48].

The second set of samples was subjected to a bacteriological exam using blood sampled from the heart, and based on cultural aspects, a culture of *Aeromonas* sp. was observed. Aeromonads are known to be of great importance both economically and medically [49]. Also, they are known to be responsible for a broad spectrum of diseases in both warm- and cold-blooded animals [50], and in the aquaculture of freshwater fish, occasionally, they can cause serious damage [51]. For the aquaculture sector, species of *Aeromonas* are of greater importance. *A. salmonicida* can cause furunculosis and *A. hydrophila, A. caviae*, and *A. sobria* can cause septicemia [52]. It is well known that these are ubiquitous, free-living, Gram-negative bacteria prevalent in aquatic habitats with a cosmopolitan distribution. They are, like many bacteria observed in aquatic environments, opportunistic pathogens that can cause heavy mortalities in farmed and feral fish [53].

The samples from the third basin were examined using blood from heart and liver tissues, resulting in a mixed culture of *Aeromonas* sp. and *Escherichia coli.* The blood sample from the heart was infected only with *Aeromonas* sp., while the samples from the liver tissue gave a mixed culture of *Aeromonas* sp. and *E. coli*. In the case of *E. coli*, although it is one of the most studied microorganisms, its presence in poikilothermic animals has been relatively understudied [54]. Some studies have documented the establishment and persistence of this organism in the rainbow trout (*O. mykiss*) intestine. At 15 °C, *E. coli* was revealed to increase in number in the fish intestine and could still be detected after 4 days [55]. In the case of the fish, *E. coli* was also used as an indicator of contamination of external origin and it was assumed that this bacteria was not normally present in the fish itself. Studies have proven that it can be detected in the intestinal tract of the fish [56], on the gills, in the muscle tissue, on the skin [57], and, in this case, even in samples harvested from the fish liver.

Real-time PCR was performed on two of the *Aeromonas* spp. strains using the NZYTech Real-time PCR Kit and protocol for *A. salmonicida*.

**Figure 18:**
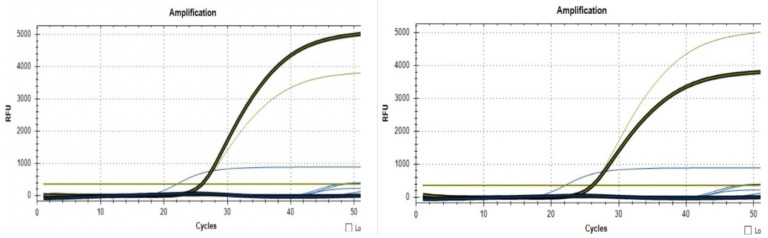
Positive results from two of the bacterial strains, confirming the species as *Aeromonas salmonicida* (Real-time PCR). The fist black line on the bottom is the baseline; The second black line is the sample going from the baseline through difrent phases: Liniar-ground phase, Exponential phase, Log-linear phase and plateau phase; The blue line is the threshold; And the yellow line that goes upwords is the positive control.

The results of qRT-PCR performed from the bacterial cultures showed 2/4 positive samples of the species *A. salmonicida*(Figure 18). The other two samples examined were negative, but being made from bacterial cultures on special media, the bacterial strains proved the presence of another *Aeromonas* species.

The qPCR Kit for *A. salmonicida* is designed for the in vitro detection of *A. salmonicida* genomes. In this study, DNA was extracted from a pure culture of Aeromonas, but this disease can cause sepsis, hemorrhages, muscle lesions, the inflammation of the lower intestine, and spleen enlargement. Because of this, DNA can be extracted from blood or organs with lesions and can be used as a safe and rapid method of diagnosis. The real-time PCR technique represents the diagnostic method with the highest sensitivity and specificity, and we are able to recommend the kit used for laboratory diagnosis.

Outbreaks produced by *A. salmonicida* tend to appear in the spring–autumn period, and mortality can be triggered by stress factors such as crowding, poor water quality, and high temperatures. The disease generally appears as a septicemic disease and is often fatal. Based on the RT-PCR results and the clinical signs displayed by the fish, it is safe to assume that a septicemic outbreak caused by *A. salmonicida*, paired with stress factors, was the main cause leading to fish mortality in this particular facility.

All the pathogens discussed so far are opportunistic; therefore, there is a strong possibility that other stress factors such as forced feeding or improper water quality might have been the primary causes that lead to the disease outbreak.

## 4. Conclusions

All the data analyzed seem to indicate an impossibility when it comes to the fish’s organisms compensating for certain stress factors such as improper water quality parameters and possibly excess feeding. This led, in time, to immunosuppression that allowed opportunistic pathogens to cause a disease outbreak with few obvious clinical signs at first. That being said, it can be strongly suggested that common bacteria normally living in water can become a significant problem if the environment is unfavorable for fish.

Also, certain lesions noticed in the fish liver can also be attributed to such pathogens, but can also be caused by toxins in the water. Regardless, lesions like the necrosis of the liver may ultimately lead to the fish’s deaths and to economic loss even if corrective measures are taken.

This case study shows that when raising fish, all factors have to be taken into account, and if problems appear, investigations should concern the fish itself and their environment [58].

Another issue to be addressed is water quality. After the outbreak, corrective measures were taken, and the water flow was greatly increased. After a few days, mortality rates dropped significantly, suggesting that it might have been a one-time water contamination. In order to find the contamination source, further investigations were required. The tests repeated six months after the incident proved a new event of pollution. The spectroscopic analyses brought more information about the chemical nature of the pollutants. Such cases can also serve in the future for identifying possible human health hazards linked to the groundwater reserves and their possible contamination. Nevertheless, the facilitated soil, air, water, air, soil, and water and precipitation circuit make it difficult to accurately assess the main cause of contamination, especially since the water source itself was strongly affected. Thus, a periodic monitoring of the entire area where both the source and the basin are located is required.

The clinical presentation corresponds to a superacute evolution of an *A. salmonicida* infection previously described in scientific literature. Knowing the exact species of *Aeromonas* ensures, in the future, effective treatment and prophylaxis, thus avoiding economic loss. After collecting all the necessary information, it was determined that the cause of death was a septicemic infection that led to a rapid and severe disease progression. This was likely due to weakened immune system responses caused by exposure to poor water quality.

Through the present study, a causal relationship was identified, at least in the sense of the aggravation of fish illness under the influence of pollution induced by the chemical compounds identified in the water. It can thus be concluded that studies on the influence of each compound on bacterial development and/or decreases in the immune response in fish are valuable assets when facing such challenges.

## Figures and Tables

**Table 1:** Water pH.

pH			
* Water Source	23 September 2022	1. Nonhomogeneous on a vertical column: 6.7–7.6 (from the top towards the bottom) 2. After homogenization: 6.2	DESIRABLE RANGE 6.5–9.5
	28 March 2023	1.Nonhomogeneous on a vertical column: 7.7–6.9 (from the top towards the bottom) 2.After homogenization: 6.8	ACCEPTABLE RANGE 5.5–10.0
** Basin Water	23 September 2022	Homogeneous: 6.2	REFERENCE [31]
	28 March 2023	Homogeneous: 7.9	

* Water source (water with a relatively low turbidity) sampled on 23 September 2022. ** Water from the basins (water with higher turbidity that contained reddish small particles that were coagulated or which partially formed a sediment) sampled on 23 September 2022.

**Table 2:** Water quality parameters.

Analysis Name	Water Source *		Basin Water **		Desirable Range	Acceptable Range	Reference
	23 September 2022	28 March 2023	23 September 2022	28 March 2023			
NH_4_^−^	10 mg/L	<10 mg/L	10 mg/L	20 mg/L	0–1 mg/L	<4 mg/L	[30]
Total hardness	25 °D/444 mg/L (>4.5 mol/m^3^)	25 °D/444 mg/L (>4.5 mol/m^3^)	25 °D/444 mg/L (>4.5 mol/m^3^)	25 °D/444 mg/L (>4.5 mol/m^3^)	50–150 mg/L	Above 10 mg/L	[34]
SO_3_^2−^	5 mg/L	10 mg/L	5 mg/L	10 mg/L	<64 mg/L	<64 mg/L	[32]
PO_4_^3−^	10–15 mg/L	10 mg/L	10–15 mg/L	10 mg/L	1 mg/L	1 mg/L	[31]
Fe^2+^/^3+^	2 mg/L	10 mg/L	10 mg/l	10 mg/L	<0.1 mg/L for fry, less than 1.0 mg/L for most fish (Ferric iron)	<0.1 mg/L for fry, less than 1.0 mg/L for most fish (Ferric iron)	[31]
Cu^+^/^2+^	<0.1 mg/L	<0.1 mg/L	<0.1 mg/L	<0.1 mg/L	<0.1 mg/L	<0.1 mg/L	[33]
Dissolved oxygen (DO)	Not measured		8.3–8.5 mg/L		6–15 mg/L	6–15 mg/L	[11]
Temperature	10–11 °C		11.6 °C–11.9 °C		7–18 °C	<22 °C	[11]

* Water source (water with a relatively low turbidity) sampled on 23 September 2022. ** Water from the basins (water with higher turbidity that contained reddish small particles that were coagulated or which partially formed a sediment) sampled on 23 September 2022.

**Table 3:** Vibrational modes of the components contained in the samples “Basin Water” and “Water Source”.

Vibrational Bands (cm^−1^)		Functional Groups and References [16,17,35,36,37,38,39,40,41,42]
Basin Water	Water Source	
3855	3855	OH that is free in alcohols OH in carboxylic acid, known to be typical carbohydrates OH in adsorbed/absorbed water or which is present as MgOH; CaOH
3739	3739	OH as SiOH (silanol); FeOH; MgOH; CaOH
3419	3419	OH that is free and bonded in alcohols NH in primary amines (including hetero-aromatic such as pyrrol), amides, aminoacids, and amino salts (NH_4_^−^) Interference with CH aromatic (the wide peak overlaps with the vibrational range of CH aromatic of 3080–3030)
2894	2894	CH aliphatic
2370	2370	Adsorbed CO_2_
2328	2328	N=O stretching in nitrites -N^+^=N stretching in diazo compounds
1873–1752	1873–1752	C=O stretching in carbonates CO_3_^2−^
1641	1651	CH bending in aromatic compounds NO_2_ stretching asymmetrically in nitrates C=O stretching in aldehydes, amides, carboxylic acids
1554–1415	1540–1420	NO stretching in nitro compounds and nitrates CNO_2_ in aromatic nitro compounds SO_2_ stretching asymmetrically in sulfones, sulfoxides, and sulfites (COO)^-^ stretching asymmetrically and symmetrically in carboxylic acids and carboxylates NH_2_, NH_3_^+^, NH_2_^+^, NH^+^ bendings in amines, amides, amino salts
1381	1387	Medium: CH bending in aldehydes; CNO_2_ in aliphatic nitro compounds Sharp, strong, small molecules; NO_2_ stretching symmetrically in nitrates
1182	-	SO_2_ stretching symmetrically in sulfones, sulfoxides, and sulfites P=O stretching in phosphates and organics R_3_P=O
1117	1117	C-NO in aliphatic nitroso compounds SO_2_ stretching symmetrically in sulfones, sulfoxides, and sulfites SiO stretching
987	987	PO stretching in phosphates
866	866	NO stretching in nitrates CNO in aromatic nitroso compounds
620	620	SiC stretching in silanol terminal groups in organic compounds

## Data Availability

Not applicable.
